# A Comprehensive Gene Co-Expression Network Analysis Reveals a Role of *GhWRKY46* in Responding to Drought and Salt Stresses

**DOI:** 10.3390/ijms232012181

**Published:** 2022-10-12

**Authors:** Pengyun Chen, Fei Wei, Hongliang Jian, Tingli Hu, Baoquan Wang, Xiaoyan Lv, Hantao Wang, Xiaokang Fu, Shuxun Yu, Hengling Wei, Liang Ma

**Affiliations:** 1 State Key Laboratory of Cotton Biology, Institute of Cotton Research of Chinese Academy of Agricultural Sciences, Anyang 455000, China; 2 State Key Laboratory of Cotton Biology, School of Life Sciences, Henan University, Kaifeng 475004, China; 3 School of Life Sciences, Zhengzhou University, Zhengzhou 450000, China

**Keywords:** cotton, transcriptomic, abiotic stresses, WGCNA, VIGS, *GhWRKY46*

## Abstract

Abiotic stress, such as drought and salinity stress, seriously inhibit the growth and development of plants. Therefore, it is vital to understand the drought and salinity resistance mechanisms to enable cotton to provide more production under drought and salt conditions. In this study, we identified 8806 and 9108 differentially expressed genes (DEGs) through a comprehensive analysis of transcriptomic data related to the PEG-induced osmotic and salt stress in cotton. By performing weighted gene co-expression network analysis (WGCNA), we identified four co-expression modules in PEG treatment and five co-expression modules in salinity stress, which included 346 and 324 predicted transcription factors (TFs) in these modules, respectively. Correspondingly, whole genome duplication (WGD) events mainly contribute to the expansion of those TFs. Kyoto Encyclopedia of Genes and Genomes (KEGG) and gene ontology (GO) analyses revealed those different modules were associated with stress resistance, including regulating macromolecule metabolic process, peptidase activity, transporter activity, lipid metabolic process, and responses to stimulus. Quantitative RT-PCR analysis was used to confirm the expression levels of 15 hub TFs in PEG6000 and salinity treatments. We found that the hub gene *GhWRKY46* could alter salt and PEG-induced drought resistance in cotton through the virus-induced gene silencing (VIGS) method. Our results provide a preliminary framework for further investigation of the cotton response to salt and drought stress, which is significant to breeding salt- and drought-tolerant cotton varieties.

## 1. Introduction

With climate change and abnormal weather events, abiotic environmental factors, such as drought and salinity, restricted the growth and yield of crops worldwide [1,2]. The hormonal pathways, signal transcription, polysaccharide content, and lipid content in the plant are easily affected by abiotic stresses [3,4]. A series of reports demonstrated that drought and salinity stresses could affect the plant’s secondary metabolites and the gene expression level by disrupting the homeostasis of the cell [5,6]. In these processes, the large amount of plant stress-resistant TFs, such as WRKY, AP2, VQ, MYB, NAC, MAPK, bZIP, and more, were detected and proved to be involved in the main pathway of the abiotic stress in Arabidopsis, rice, soybean, maize, cotton, and more [7,8,9,10]. The microarray analysis and RT-PCR results provide WRKY, ERF, and JAZ genes as potential markers of tolerance to salt stress in cotton [11]. By comparing the transcriptomics of the two cultivars*, GhERF12* was identified as involving salinity tolerance during the early development of cotton [12]. By analyzing the long-reads RNA sequencing in cotton, the TFs were found to widely participate in the complex nature of salt stress tolerance mechanisms [13]. Moreover, the expression of hormone-related genes and salt stress-related genes in *GaJAZ1* transgenic cotton were reprogrammed [14]. Furthermore, other research also provided the hormones and TFs important to the adaptability of cotton to abiotic stress [15,16,17]. Therefore, TFs played a central role in plant tolerance and adaptability.

As indispensable TFs, WRKY proteins contain a conservative WRKY domain and are widely involved in plant multi-abiotic stresses via various hormone signal transcription pathways [18,19,20,21]. WRKY proteins have the function of regulating other stress-related genes by combining W-box and *cis*-element (TGACC (A/T)) [18,19]. In *Arabidopsis*, *AtWRKY22*, *AtWRKY25*, *AtWRKY33*, *AtWRKY46,* and more, could negatively or positively regulate resistance against various abiotic stress, such as drought and salt stresses [22,23]. In soybean, overexpression of *GmWRKY12* could enhance drought and salt tolerance [24]. 

In cotton, WRKY proteins also could play an important role in abiotic resistance. In *G.aridum*, *GarWRKY5* was found involved in salt stress response related to activating the hormone signaling pathway [25] Similarly, *GhWRKY6-like* was reported as a negative regulator in response to salt stress via the ABA signaling pathway in cotton, and *GhWRKY21* plays a role in the drought-induced ABA signaling pathway [26,27]. *GhWRKY33* was found as a negative regulator involved in the drought stress in cotton [28]. *GhWRKY41* might be a positive regulator of stomatal closure, and by regulating reactive oxygen species, enhance the plant tolerance to stress [29]. *GhWRKY91* was also identified as a hub factor in the drought stress response in cotton [30]. WRKY proteins could also be involved in the process of leaf senescence. Overexpression of *GhWRKY17* in *Arabidopsis* enhanced the plant’s susceptibility to leaf senescence [31]. *GhWRKY27* could positively regulate leaf senescence via interaction with *GhTT2* and binding to the promoters of *GhCYP94C1* and *GhRipen2–2* [32]. In addition, WRKY TFs were also involved in fiber initiation and elongation [33]. 

WGCNA is proven to be an effective method for identifying cluster gene modules and hub genes in various crops [34,35]. In maize, the WGCNA method was used to exploit multiple traits to determine core modules and hub genes [36]. In other research on cadmium resistance, 22 regulatory modules were identified in maize [37]. Furthermore, there were lots of studies on the identification of hub genes related to fiber quality and resistance to stress response in cotton. In a recent report, Zou et al. reported five specific modules pertaining to fiber development at different growth stages [38]. Cheng et al. indicated 574 TFs and 936 hub genes related to cotton seedling cold resistance [16]. Moreover, a meta-analysis of cotton transcriptomics identified some hub genes, including *RETICULON-like 5* (*RTNLB5*) and *PRA1*, involved in regulating stress responses [39]. At the seedling stage, a comprehensive analysis of two cotton genotype transcriptomics recovered the plant MAPK signaling pathway and diterpenoid biosynthesis involved in response to salt stress [40]. Additionally, a detailed analysis of the evolution and abiotic stresses in *G. thurberi*, *G. klotzschianum*, *G. raimondii*, and *G. trilobum* might provide available gene resources underlying a multi-abiotic-resistant cotton breeding strategy [41]. In *G. arboretum*, various tissue and stress-related transcriptomics were used to construct co-expression networks with over 500,000 pairs of edges and 33,413 nodes [42]. In addition, ccNET (http://structuralbiology.cau.edu.cn/gossypium/) (accessed on 20 August 2020), COTTONOMICS (http://cotton.zju.edu.cn/index.htm) (accessed on 20 August 2020), MaGenDB (http://magen.whu.edu.cn) (accessed on 20 August 2020) et.al websites were useful to the study of the co-expression functional analysis in cotton [43,44]. Therefore, the WGCNA can be used as a reliable method to estimate the gene function and further apply it to cotton breeding.

Cotton is one of the most important industrial crops, and it is grown for its elite fibers and oil for industries worldwide. However, the drought and salinity seriously limited the production and quality of cotton [45]. Thus, it is indispensable to mine the genes related to cotton’s drought and salt stress. The completion and continuous update of cotton genome sequencing and transcriptome data made it possible to identify and exploit key genes related to cotton breeding [46,47,48]. In this study, the DEGs and WGCNA methods were performed to analyze the expression profile of previous transcriptomics data related to salinity and PEG-induced drought tolerance. Four and five co-expression modules related to drought and salt stress were constructed. Transcription factors, including WRKY, MYB, bHLH, and ERF proteins, were widely identified in these modules, distributed in every chromosome, and the WGD events mainly contributed to their expansion. In addition, a hub *GhWRKY46* (*GH_D07G1505*) was isolated, which is located in the nucleus, and qRT-PCR assays indicate that *GhWRKY46* responded to salt and PEG6000 stress resistance in cotton. Furthermore, silencing *GhWRKY46* decreased the salinity and PEG6000 tolerance in cotton, suggesting that *GhWRKY46* played a critical role in regulating the salinity and PEG-induced drought tolerance. This study will benefit the development of abiotic-resistant varieties in cotton breeding.

## 2. Results

### 2.1. Transcriptome Sequences and Differential Expression Analysis

The transcriptome sequence related to the drought and salt treatment was downloaded from the SRA database to identify genes related to abiotic stress in cotton. A total of 1754.18 million raw reads and 1678.94 clean reads were obtained, with an average of 26.23 million reads per sample. For each sample, the GC content of the clean reads was from 43% to 45%, and the high-quality reads that mapped to the *G.hirsutum* reference genome were ranging from 97.11% to 98.93% (Table S1). 

**Figure 1:**
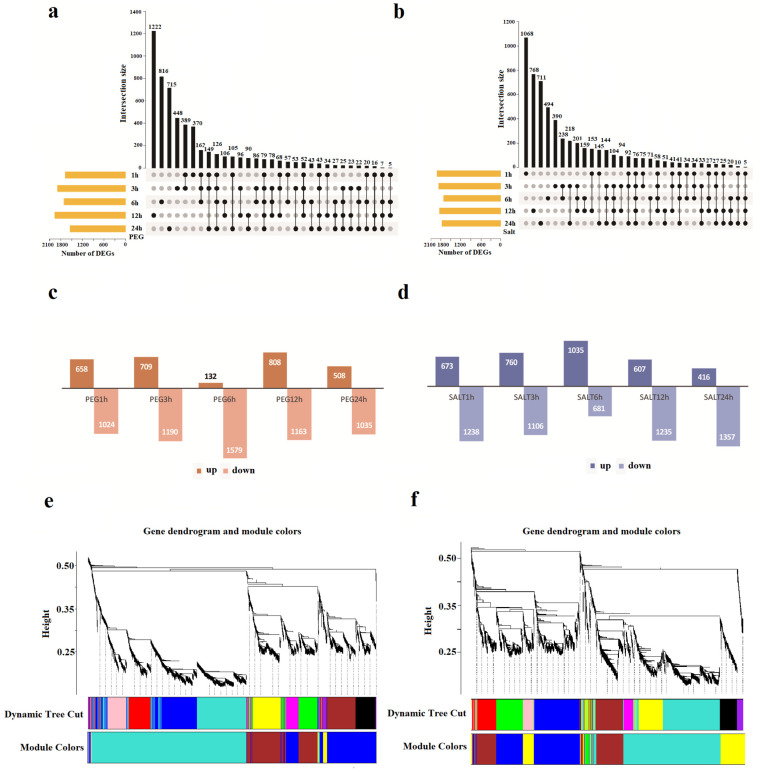
Expression dynamic changes, as well as comparative and module detection analysis of DEGs between the drought and salinity stresses. (**a**), Upset chart of the DEGs number in different time stages under PEG treatment. (**b**), Upset chart of the numbers of DEGs in different time stages under salinity treatment. (**c**), Numbers of DEGs up-regulated and down-regulated under PEG treatment. (**d**), numbers of DEGs up-regulated and down-regulated under salinity treatment. (**e**), Gene co-expression modules of the PEG treatment. The columns represent modules and different colors represent different modules. (**f**), Gene co-expression modules of the salinity treatment. The columns represent modules and different colors represent different modules.

In this study, the data sets include the transcript with expressed levels, with FPKM > 1 in at least three samples. A total of 25,655 and 24,542 genes related to drought (PEG) and salinity (NaCl) stresses were obtained, respectively (Figure 1a,b; Table S3). The largest number of DEG (1899) was identified in PEG at 3 h, and the smallest number of DEGs (1543) was identified in PEG at 24 h (Figure 1a,c; Table S3). However, under salt stress, it was estimated that the amount of DEG was the highest (1911) at 1 h, and the lowest amount of DEG (1711) was obtained at 6 h (Figure 1b,d; Table S3). As is depicted in the Venn diagram, 3494 DEGs were detected in the two stresses (Table S3; Figure S1).

### 2.2. Gene Co-Expression Construction and Analysis

To reveal the potential regulatory pathways for resistance to drought and salt stress in cotton, we constructed the co-expression modules through the WGCNA method. In this study, we selected the weight value β = 18 to construct the scale-free networks, describing different modules with different colors and merging similar modules. These modules were defined as clusters of highly interconnected genes, and genes within the same cluster have high correlation coefficients and potential functional relations. Four co-expression modules related to the PEG treatment were constructed. The turquoise module (2690) was with the maximum count, and the yellow module was with the minimum (119) (Figure 1e; Table 1). Under the PEG treatment, the range of the up-regulated DEGs in different modules was from 9 (7.56%, the yellow module) to 461 (17.14%, the turquoise module), and the down-regulated number varied from 15 (12.61%, the yellow module) to 196 (7.29%, the turquoise module). Furthermore, clustering analysis suggested five co-expression modules related to salinity treatment. The largest module was the turquoise module, which contained 1900 genes, and the smallest module was the green module, including only 64 genes (Figure 1f and Table 1). The up-regulated DEGs in the different modules varied from 4 (6.2%, the green module) to 265 (13.95%, the turquoise module), while the down-regulated varied from 14 (4.55%, the green module) to 266 (19.57%, the blue module) (Table 1).

### 2.3. The Characteristics of the Genes Identified in the Networks 

**Figure 2:**
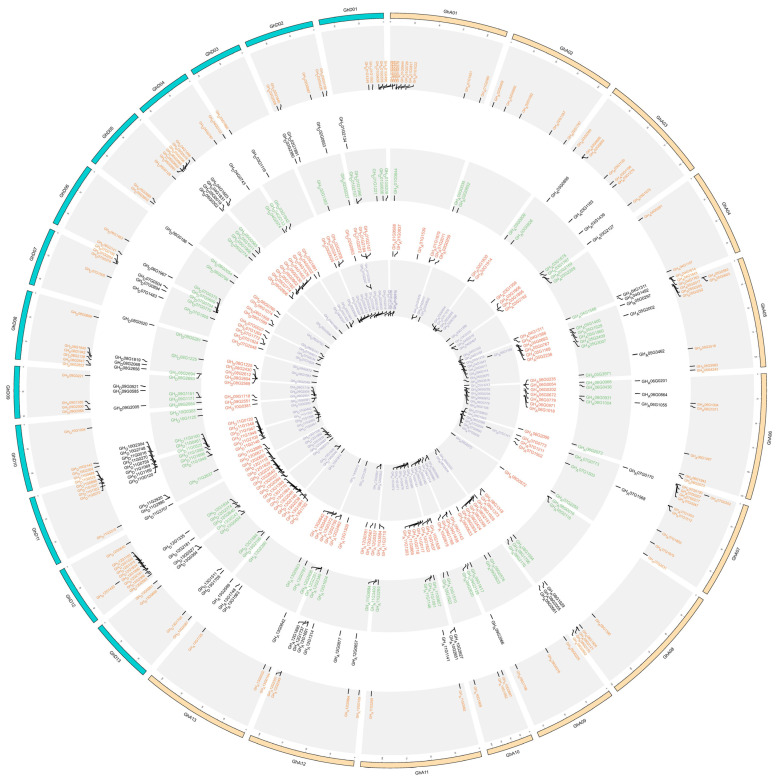
Distributions of the predicted TFs on chromosomes of the *G.hirsutum*. The gene color in the blue module under PEG stress is orange, while in the turquoise module is red. The gene color in the brown module under salinity stress is black, while in the blue module and turquoise is green and purple, respectively.

We next predicted the TFs in those modules through the PlantTFDB website. In the PEG-treated module, 6 (5.04%), 75 (7.81%), 133 (11.20%), and 132 (4.91%) TFs were identified in the yellow, brown, blue, and turquoise modules, respectively (Table S1). The largest proportion of the TFs in the PEG-treated module was MYB (14.94%), followed by WRKY (9.48%), bHLH (8.62%), ERF (8.62%), NAC (7.76%), bZIP (4.49%), and C3H (3.45%) (Table S4). Similarly, 3 (4.54%), 57 (8.06%), 70 (7.38%), 97 (5.11%), and 97 (7.14%) TFs were found in the green, yellow, brown, turquoise, and blue salt-treated modules, respectively (Table 1). In subsequent analysis, some important TFs, including MYB (12.04%), WRKY (8.95%), ERF (8.02%), bHLH (7.40%), NAC (7.40%), and bZIP (6.79%), were predicted from the salt stress modules (Table S4). Furthermore, we found that these TFs were distributed in twenty-six cotton chromosomes and three scaffolds (Figure 2; Table S5). Many TFs were distributed at both ends of each chromosome, which corresponded to the position of the telomere. Moreover, most of the TFs were distributed in the D01 chromosome (35/6.48%), followed by A05 (29/5.37%), A08 (27/5%), A11 (27/5%), D12 (28/5.3%), and A12 (26/4.81%), while D04 (11/2.03%) and A04 (12/2.22%) contained a few genes (Figure 2; Table S5). Among those identified TFs, 148 (27.4%) genes had no intron, 97 (17.96%) genes had one intron, and 2 genes (*GH_A10G0038* and *GH_A13G2589*) contained 17 introns (Table S5), and the length of those TFs CDS (coding sequence) ranged from 234 to 5172 bp. 

We further analyze duplication events of those TFs to explore their expansion mechanism. In the modules related to PEG and salt treatment, the duplication type of these TFs was identified (Table 2). For the PEG-treatment modules, there were 1, 3, and 132 TFs related to dispersed, tandem, and WGD events in the blue module, respectively; 1, 3, and 128 TFs were detected relating to dispersed, tandem, and WGD events in the turquoise module, respectively (Table S6). Meanwhile, in the salt stress modules, 2, 2, and 66 TFs were related to dispersed, tandem, and WGD events in the brown module, respectively. In the blue module, 1, 3, and 93 TFs might expand by dispersed, tandem, and WGD events, respectively; 2 dispersed and 95 TFs were detected relating to dispersed, tandem, and WGD events in the turquoise module, respectively (Table S6). Therefore, WGD events mainly contributed to the expansion of identified TF in the modules related to salt and PEG-induced drought stress.

### 2.4. Candidate Module Identification and Functional Analysis

With the selected correlation value of the |r| > 0.7, four modules were found in the PEG stresses, including the yellow module (r = 0.8, *p* = 0.1) at 6 h, the blue module (r = 0.85, *p* = 0.07) and the brown module (r = 0.83, *p* = 0.09) at 12 h, and the turquoise modules with a negative correlation (r = −0.72, *p* = 0.2) at 24 h (Figure S2). For the salt stress, the brown module (r = 0.78, *p* = 0.1) and turquoise module (r = 0.71, *p* = 0.2) were positively correlated at 12 h, while the green module (r = −0.9, *p* = 0.04), yellow module (r = −0.85, *p* = 0.07), and blue module (r = −0.71, *p* = 0.2) were negatively correlated at 1 h, 6 h, and 24 h (Figure S2). 

**Figure 3:**
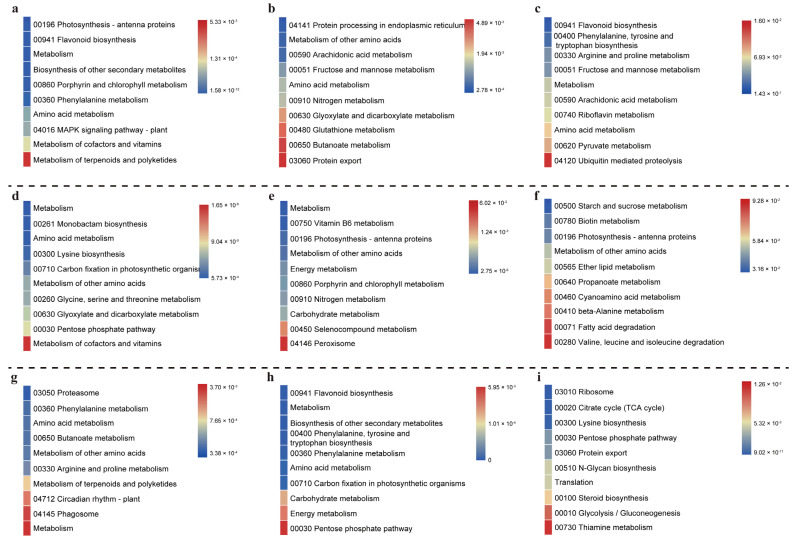
The KEGG pathway enrichment statistics in the modules of PEG and salinity treatments. (**a**–**d**), the top 10 KEGG pathways in the blue, brown, yellow, and turquoise modules of PEG treatments. (**e**–**i**), the top 10 KEGG pathways in the blue, green, brown, yellow, and turquoise modules of salinity treatments.

To uncover the potential function in the above modules, we performed KEGG enrichment analysis in different modules in this study. KEGG analysis of the four modules related to PEG treatments indicated that the potential pathways were enriched in the metabolism of terpenoids and polyketides, protein export, ubiquitin mediated proteolysis, and metabolism of cofactors and vitamins (Figure 3a–d). Among the pathways related to salt treatment, the metabolism pathway is the vast majority pathway, such as peroxisome, valine, leucine and isoleucine degradation, pentose phosphate, and thiamine metabolism (Figure 3e–i). Additionally, GO enrichment analysis was also performed in this study (Table S8). In the PEG treatment analysis, the GO enrichment in the blue module suggested those genes were mainly involved in transferase activity, transferring hexosyl groups, oxidoreductase complex, and oxidoreductase complex. The genes related to cell metabolism, macromolecular metabolic process, heat shock protein, and water absorption were found in the brown module and are essential to cotton drought adaptation. At the same time, the turquoise module was enriched in peptidase activity, aspartic peptidase activity, oxidoreductase, ion transport, defense mechanisms, and other multi-biological processes. Correspondingly, GO analysis in the salt treatment of the blue module was related to material transport, transmembrane transport, ion steady state, and cell walls. Additionally, in the turquoise module, the electronic signal transmission, abscisic acid reaction, reactive oxygen response, and secondary metabolites were enriched. The gene in the brown module is mainly related to cell stimulation and toxicity response, such as peroxides and superoxide. Furthermore, in the yellow module, the genes were enriched in the organic acid metabolic process, carboxylic acid metabolic process, monocarboxylic acid biosynthetic process, and fatty acid biosynthetic process; and the genes in the green module were enriched in the chloroplast, plastid, anatomical structure development, and developmental process (Table S8). Detailed function annotations make it better to understand the function of distinguishing gene lists in different modules through WGCNA and multiple gene function annotation analysis.

### 2.5. Identification of Hub Genes and Gene Expression Assays

**Figure 4:**
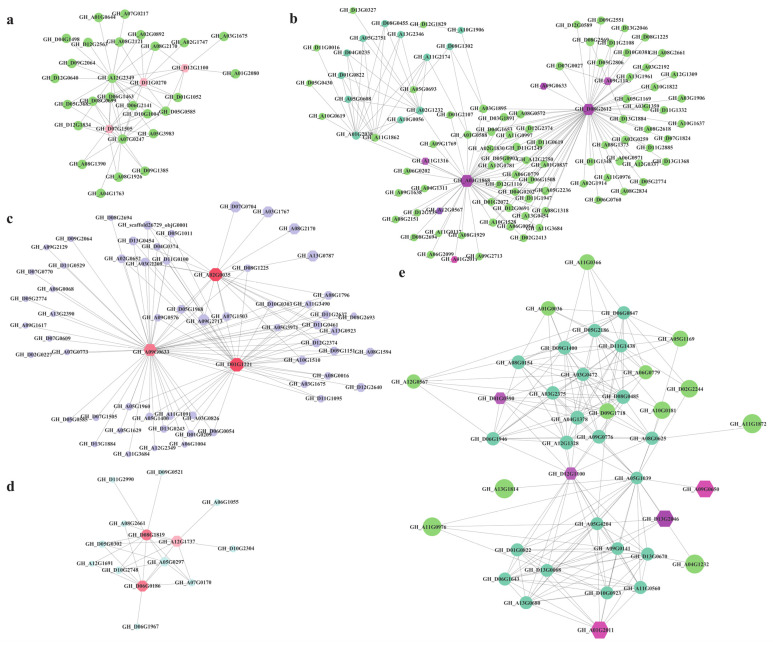
Gene networks of hub genes for significant co-expression modules. The genes with higher connectivity in the corresponding networks are shown with larger circle sizes. The size of the node circle is positively correlated with the degree of genes that it partners in interaction. (**a**), The PEG stress-related co-expression network genes in the blue module. (**b**), The PEG stress-related co-expression network genes in turquoise module. (**c**), The salinity stress-related co-expression network genes in the blue module. (**d**), The salinity stress-related co-expression network genes in the brown module. (**e**), The salinity stress-related co-expression network genes in the turquoise module.

The correlation network and hub genes were further constructed and identified by preforming the CytoHubba package in Cytoscape software (Figure 4). In the gene expression regulation network, hub genes might interact with other genes through interaction or regulation. In modules related to PEG stress, three hub TFs were identified in the blue module, including WRKY (*GH_D07G1505*), HAT (*GH_D11G0270*), and SCL (*GH_D12G1100*) (Figure 4a); seven hub TFs were also identified in the turquoise module, including MYBS (*GH_A09G0633*), MYB (*GH_A09G1143*), WHIRLY (*GH_D08G2612*), bHLH (*GH_A11G1316*), BBX (*GH_A03G1868*), and COL (*GH_A12G0567* and *GH_A01G2011*) (Figure 4b). Moreover, in the module related salt stress, three hub TFs were identified in the blue module, which contained WRKY (*GH_A02G0035*), MYBS (*GH_A09G0633*), and REM16 (*GH_D01G1221*) (Figure 4c). In the brown module, three hub genes were identified, including KAN2 (*GH_D08G1819*), HSFA8 (*GH_A12G1737*), and RAP2 (*GH_D06G0186*) (Figure 4d). In the turquoise modules, a total of 5 hub TFs, including COL genes (*GH_A01G2011* and *GH_A09G0650*), a GRAS gene (*GH_D12G1100*), P450 (*GH_D01G0390*), and MADS-box gene (*GH_D13G2046*) were identified (Figure 4e). These mined modules might provide a potential functional connection between the hub genes and other functional genes.

**Figure 5:**
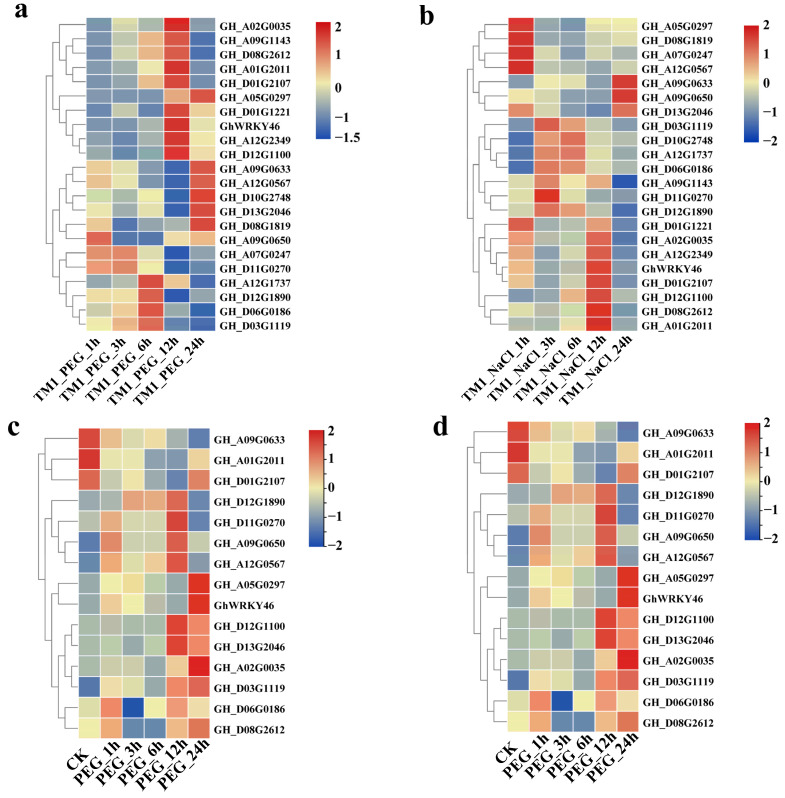
Expression profiling of 15 selected hub TFs; (**a**,**b**), the expression of 15 selected hub TFs in PEG and salinity in transcript profiling, respectively; (**c**,**d**), qRT-PCR analysis of 15 selected hub TFs in PEG and salinity, respectively. *GhUBQ7* served as the reference gene.

Next, 15 hub TFs were selected for further qRT-PCR analysis to confirm their potential function. The qRT-PCR results suggest that TFs were involved in response to stress resistance at different stages in cotton (Figure 5; Table S7). Some selected genes had a high expression at 12 h and 24 h under the abiotic stress, especially the genes including WRKY TFs (*GH_A02G0035* and *GH_D07G1505*), MADS-box TF (*GH_D13G2046*), and COL TFs (*GH_A01G2011* and *GH_D01G2107*) (Figure 5a; Table S7). Almost all of the related genes’ promoter sequences contain more than one WRKY binding site, which might be regulated by the WRKY proteins (Table S9). Therefore, we speculate that these hub genes might play a role in responding to salt and PEG-induced drought stress through participating in the pathway related to WRKY proteins.

### 2.6. Silencing of GhWRKY46 Decreased Salt and PEG-Induced Drought Tolerance

**Figure 6:**
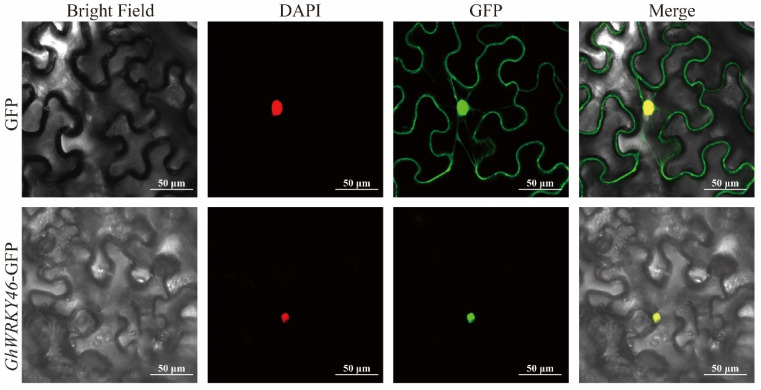
Subcellular localization of the fused *GhWRKY46* in tobacco leaf cells. The 121-GFP was used as the control. Nuclear DAPI staining was expressed in the same cell. The scale bar is 5 µm.

In order to verify the accuracy of data analysis and the function of the hub gene, we selected *GhWRKY46* for further functional analysis. The qRT-PCR results show that *GhWRKY46* participated in the salt and PEG-induced drought responses. As a homologous gene of *AtWRKY46* (*AT2G46400*) in Arabidopsis (Figure S3), *GhWRKY46* contained a conserved WRKY domain and had three exons and two introns (Figure S3). Next, by performing the subcellular localization assay, we found that *GhWRKY46* can be transported into the nucleus of *N. benthamiana* cells, which suggests that *GhWRKY46* might perform its functions in the nucleus (Figure 6).

**Figure 7:**
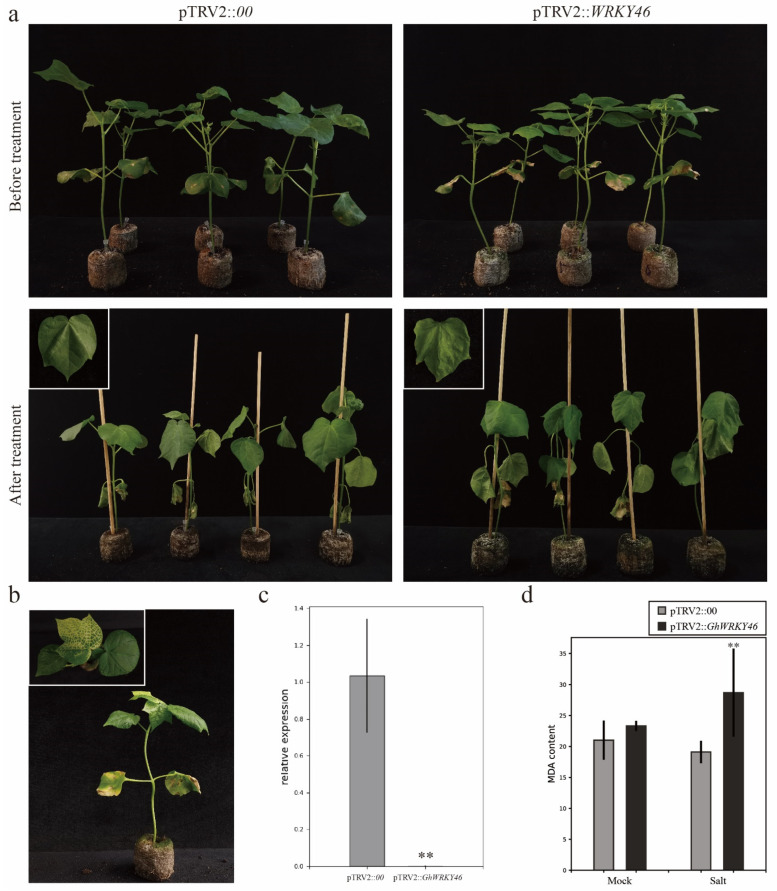
Silencing *GhWRKY46* via VIGS decreases salt tolerance in cotton. (**a**), Phenotype of pTRV2::*GhWRKY46* plants under 400 mM NaCl treatment. (**b**), Albino phenotype after pTRV2::*CLA1* silencing. (**c**), Relative expression of *GhWRKY46* in pTRV2::*00* and silencing pTRV2::*GhWRKY46* plants via qRT-PCR analysis. (**d**), The MDA content of pTRV2::*00* and pTRV2::*GhWRKY46* after salinity stress. Error bars represent the standard deviation of three independent biological replicates (** *p* < 0.01 Student’s *t*-test).

**Figure 8:**
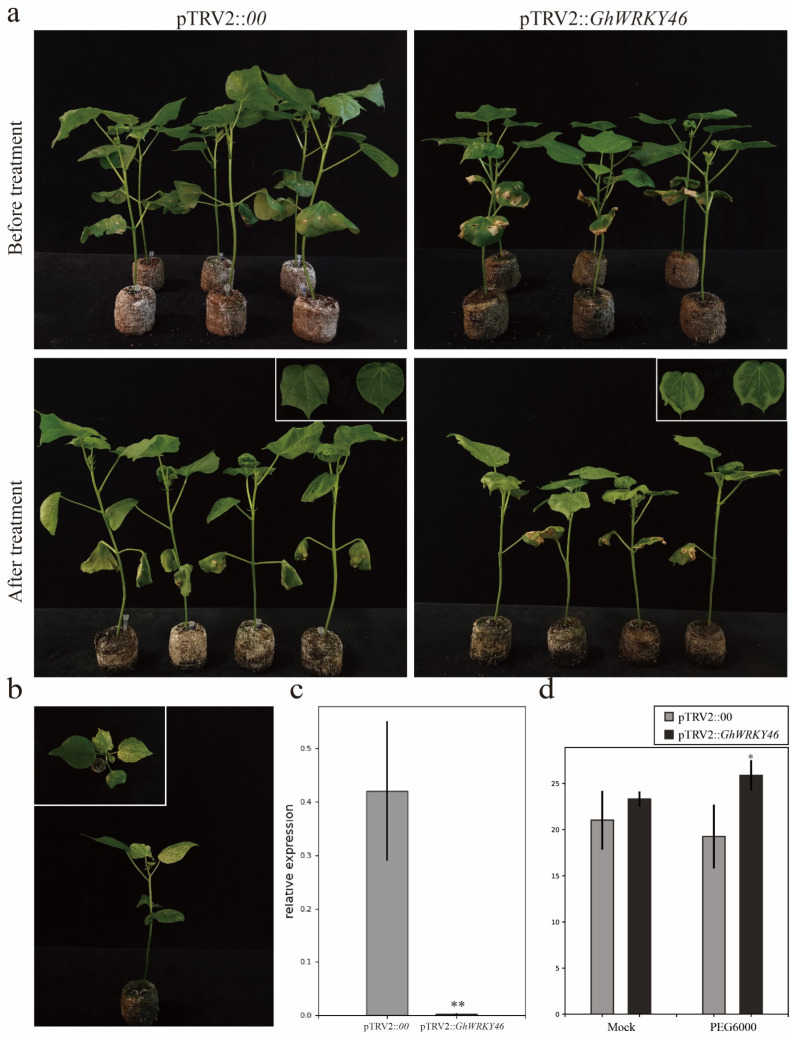
Silencing *GhWRKY46* via VIGS decreases PEG6000 tolerance in cotton. (**a**), Phenotype of pTRV2::*GhWRKY46* plants under PEG6000 treatment. (**b**), Albino phenotype after pTRV2::*CLA1* silencing. (**c**), Relative expression of *GhWRKY46* in pTRV2::*00* and silencing pTRV2::*GhWRKY46* plants via qRT-PCR analysis. (**d**), The MDA content of pTRV2::*00* and pTRV2::*GhWRKY46* after salinity stress. Error bars represent the standard deviation of three independent biological replicates (* *p* < 0.05, ** *p* < 0.01 Student’s *t*-test).

The cotton lines transformed with the pTRV2::*CLA1* show an albino phenotype, indicating that the VIGS experiment was successful (Figure 7b and Figure 8b). The expression level of *GhWRKY46* in the silent plants was significantly lower than in the pTRV2::*00* plants (Figure 7c and Figure 8c). Three weeks later, the silenced and control plants were irrigated with a 400 mM NaCl solution. The results indicate that the silenced plants show a salt-sensitive phenotype after treatment for two days compared with the control (Figure 7a). The MDA content in pTRV2::*GhWRKY46* plants was significantly higher than that in pTRV2::*00* plants (Figure 8d). For the PEG6000 treatment, we also found that the wilting was more apparent in the leaves of pTRV2::*GhWRKY46* plants than in the pTRV2::*00* plants under the PEG6000 treatment (Figure 8a). Additionally, the MDA content was higher than that in the control plants (Figure 8d). Our results prove that silencing of the *GhWRKY46* can reduce cotton tolerance to salt and PEG-induced drought stresses.

## 3. Discussion

Cotton is an important economic crop with natural abiotic resistance and is widely planted in the world. Abiotic stresses, including drought and salinity stress, affected the cotton’s growth and restricted the planting area in the world [1,2]. Therefore, understanding the complicated potential mechanisms and serious pathways, including genes related to hormone signal transcription, gene expression, peptide, and physiological indicators, will better explore the resistance mechanism and improve the existing crop varieties [3,5,6]. Studies in Arabidopsis, rice, cotton, and more, show that transcriptomics sequencing is a fast and effective method to obtain candidate genes and predict functional regulation pathways [44,49,50,51,52,53]. Here, we performed a comprehensive analysis of the transcriptomics data under the PEG and salt stresses in cotton and explored the potential network in upland cotton.

### 3.1. DEGs, Co-Expression Network, and Polyploidization Event Analysis

The R/edgeR and R/WGCNA packages were widely used to explore DEGs and core gene-related traits or stress-tolerant genes and related mechanisms in plant transcriptomics. In our study, a large number of drought-responsive and salinity-responsive genes were identified, and their up-regulated or down-regulated DEGs were clearly displayed (Figure 1c,d and Figure S1; Table S3). These results indicate that induced gene expression, at different periods, is various, and different gene sets were activated in response to salt and drought stress. In the current study, we found 25,655 and 24,542 DEGs in the drought and salinity stresses, and 3494 DEGs were detected in both DEGs (Figure S1; Table S3). Followed by the WGCNA analysis, four modules were found in PEG stress and five modules in salinity stresses (Figure 1, Table 1). By performing the TFs prediction, WRKY, MYB, bHLH, ERF, and more, domain-containing genes were found in various modules. 

The polyploidization event significantly contributed to the plant’s adaption to environmental changes and led to the expansion of plant genomes and gene numbers [54,55]. Given that the WGD event is the main factor that doubles the plant genome and promotes stress resistance adaptation, research on those genes was essential to uncover the potential regulatory mechanism [56]. We further found that the TFs expanded by the WGD event were counted at 97.36% and 96.21% in modules related to PEG and salinity stresses, respectively (Figure S1; Table S3). Additionally, the WRKY domain-containing genes in this study were displayed as 28 (10.56%) and 27 (10.23%) in the PEG and salinity stress modules, respectively. Therefore, we speculated that WRKY proteins might be the main factor in abiotic stress resistance in cotton.

### 3.2. Gene Enrichment Analysis and Candidate Gene Identification 

Antioxidant and transporter activity are essential protective mechanisms that protect the plant from abiotic stress. A study on the salt-tolerant genotype, Zhong9807, showed that GO terms were mainly enriched in catalytic activity, transporter activity, and antioxidant activity, and the KEGG were mainly enriched in hormone synthesis related, ROS related, and hormone signal transduction related pathways [13]. In addition, genes associated with “response to oxidative stress,” “antioxidant activity,” and “peroxidase activity” were significantly enriched in salt-tolerant and sensitive cotton genotypes [12]. Other abiotic stress research also indicated that “signal transduction” and “secondary metabolite biosynthesis”, and more, pathways are essential to plant growth and adaptable development [13,57]. In the current study, in order to understand the function of PEG-related and salinity-related genes in cotton, the different modules’ gene list was also analyzed by KEGG and GO enrichment. For both drought and salinity stresses, our study found enrichment of the KEGG pathway associated with “terpenoids and polyketides”, “protein export”, “ubiquitin mediated proteolysis”, “metabolism of cofactors and vitamins”, “fatty acid degradation”, “phagosome”, “carbohydrate metabolism”, “circadian rhythm” and “thiamine metabolism” (Figure 3). Moreover, “transferase activity”, “cell wall”, “inorganic ion homeostasis”, “photosystem”, “biological regulation”, “signal transduction”, “hormone signaling (abscisic acid and ethylene)”, and more, were also widely identified in GO terms (Table S8). Taken together, signal transport and hormonal pathway responses were related to the PEG and salinity stress in cotton, which corresponded to previous research.

The above analysis indicates that the hub gene might potentially regulate the abiotic stresses, especially the TFs, as the candidate stress-related genes set (Tables S3 and S4). As key regulators of abiotic stresses, WRKY TFs play a critical role in broad stress adaptation. Previous studies prove that *GhWRKY17*, *GhWRKY21*, *GhWRKY27*, *GhWRKY33*, *GhWRKY41*, *GhWRKY70*, and other GhWRKYs could regulate the resistance to salt, drought, verticillium wilt, and other abiotic stresses, respectively [27,28,29,31,32,58]. Moreover, other essential regulators of stress tolerance TFs, including MYB, ERF, bHLH, NAC, and bZIP, were also identified and selected to construct the co-expression network (Figure 4; Table S4). In addition, the analysis of their promoter sequence indicated a complicated regulation in stress tolerance in cotton (Table S9). Altogether, identification of the TFs in stresses and duplication analysis shed a new light on the significant contribution to cotton adaption in multi-abiotic stresses.

### 3.3. Silencing GhWRKY46 Enhanced the Sensitivity to Salinity and Drought in Cotton

Previous studies show that *AtWRKY46*, the homolog gene of *GhWRKY46*, plays dual roles in regulating plant response to drought and salt stresses. It interacts with *AtWRKY50/70* as a signaling component involved in BR-regulated growth and drought responses [23]. Here, we made a comprehensive co-expression analysis and observed a hub gene, *GhWRKY46*, and we further found that *GhWRKY46* could respond to salt and PEG6000 treatment. In addition, the silencing of *GhWRKY46* enhanced sensitivity to salinity and drought in cotton (Figure 7a and Figure 8a). These results indicate that *GhWRKY46* might participate in regulating salinity and drought stress in cotton. Previous studies show that the MDA content was related to oxidative stress and redox signaling, particularly in plant abiotic stresses, and an indicator of ROS-dependent cell damage [59]. Our results here show that MDA content experienced a significant change in the *GhWRKY46* silencing plants under the treatment of salt and PEG6000, indicating that *GhWRKY46* may contribute to salt and drought stress response through regulating ROS scavenging (Figure 7d and Figure 8d).

## 4. Materials and Methods

### 4.1. Acquisition and Comparison Analysis of Cotton Transcriptome Data

A transcriptome project (PRJNA490626) that contained 32 transcriptomes from previous research from the NCBI SRA (Sequence Read Archive) database was downloaded, which related to the cotton seedling treated with sodium chloride and PEG stress (Table S1) [46]. FastQC (https://www.bioinformatics.babraham.ac.uk/projects/fastqc/) (accessed on 16 July 2020) and Trimmomatic (version 0.3.9) were used to perform sequencing quality evaluation and low-quality read filtration, respectively [60]. HISAT2 was used to build the *G.hirsutum* genome (ZJU2.1 version) index file [46,61]. Samtools (version 1.9) and featureCounts (version 1.5.3) were used for data format conversion and calculating the gene FPKM (Fragments per Kilobase per Million) value, respectively [62,63].

### 4.2. DEGs Analysis and Gene Co-Expression Construction 

The DEGs of PEG and salinity treatments post of 1 h (h), 3 h, 6 h, 12 h, and 24 h were identified by edgeR (R version 3.10) [64]. Genes with | logFC | > 1 and *p*-value < 0.05 were selected as DEGs in this study. The WGCNA (version 1.69) package was used to construct the weighted gene co-expression network, divide the relevant modules, and select hub genes [65]. The weight value was calculated by pickSoftThreshold in the WGCNA package, and β = 18 was selected to perform power processing to obtain a scale-free adjacency matrix on the original scaled relationship matrix. The topological disparity matrix (dissTOM = 1-TOM) and the dynamic shearing algorithm were used to classify gene clustering and the module division. The minimum number of genes in the module is 30 (minModuleSize = 30), and the merge threshold of similar modules is 0.25 (cutHeight = 0.25). Furthermore, the CytoHubba package in the Cytoscape software (version 3.7.2) was used to visualize the network in the modules [66,67].

### 4.3. Gene Enrichment, TFs, and Duplication Prediction

The eggNOG-Mapper software was used for gene annotation through the matches of the protein sequences of *G.hirsutum* (ZJU2.1 version; http://cotton.zju.edu.cn/index.htm) (accessed on 11 August 2021) [46,68]. ClusterProfiler (version 3.14.13) was used for GO and KEGG enrichment analysis [69]. The protein sequences were submitted to the PlantTFDB database (http://planttfdb.cbi.pku.edu.cn/) (accessed on 13 August 2021) to predict the TFs. The *cis*-elements and motifs in the promoter sequences were searched and analyzed by the PlantCare website (http://bioinformatics.psb.ugent.be/webtools/plantcare/html/) (accessed on 14 August 2021) and the MAmotif software (https://github.com/shao-lab/MAmotif) (accessed on 12 September 2021), respectively [70,71]. The duplication events were analyzed by the MCScanX software with the default parameters (http://chibba.pgml.uga.edu/mcscan2/) (accessed on 16 August 2020) [72].

### 4.4. RNA Extraction and qRT-PCR Analysis

Upland cotton cultivar TM-1 seeds were grown in the growth room. Seedlings with uniform growth at the three-leaf stage were treated with 400 mM PEG and 400 mM NaCl, respectively. Leaf samples were collected at 0 h, 1 h, 3 h, 6 h, 12 h, and 24 h after treatment and rapidly saved in liquid nitrogen and stored at −80 °C in the refrigerator. The RNA extraction kit (Polysaccharides & Polyphenolics-rich, DP441) and the Takara reverse transcription kit (Mir-X TM MIRNA First-Strand Synthesis Kit) were used for RNA extraction and RNA reverse transcription. The Roche LightCycler 480 System (Roche, Germany) with the Cowin Bio and UltraSYBR One-Step Fluorescence Quantitative PCR Kit (UltraSYBR One-Step RT-qPCR Kit) were used to perform qRT-PCR. The *GhUBQ7* gene was selected as the internal reference gene. Primers for qRT-PCR are shown in Table S7. The reaction procedure is: 95 °C for 10 min preheat denaturation, 95 °C for 5 s, 60 °C for 15 s, 72 °C for 10 s, 40 cycles; melting curve programs are: 95 °C for 15 s, 60 °C for 1 min, 95 °C for 15 s; reaction system is: 2xUltra SYBR Mixture 10 μL, forward primer (10 µmol L^−1^) 0.6 μL, reverse primer (10 µmol L^−1^) 0.6 μL, cDNA 0.8 μL, and ddH2O 8 μL. Three biological replicates were taken for each sample, and three independent experiments were performed. The results were calculated using the relative quantitative method 2^−ΔΔCt^ [73].

### 4.5. Subcellular Localization

The full-length CDS of *GhWRKY46* was amplified from the upland cotton cultivar TM-1 and cloned into the pBI121-GFP vector. The leaves of six-week-old *N. benthamiana* leaves were used to inject pBI121-GFP, DAPI, and *GhWRKY46*-GFP, respectively. After the injection, the *N.benthamiana* plants were treated with dark for 24 h, then exposed to light treatment for 48 h. Observations under the laser confocal microscope were recorded.

### 4.6. Virus-Induced Gene Silencing of the GhWRKY46 in Cotton

A 300-bp fragment of *GhWRKY46* was amplified and cloned into the pTRV2 (pYL156) vector to produce pTRV2::*GhWRKY46* constructs, and the primers were listed in Table S2. The recombinant construction vector was transformed into the Agrobacterium tumefaciens strain LBA4404. The cotyledons of TM-1 cotton seedlings were used to inject an equal amount of Agrobacterium expressing the vectors, including pTRV2::*00* (empty vector), pTRV2::*GhWRKY46*, pTRV2::*CLA1* (positive control), and pTRV1 (pYL192, helper vector), as previously described [12,25,31]. Three weeks later, the plants with pTRV2::*00* and pTRV2::*GhWRKY46* were subjected to salt and PEG6000 stress. The malondialdehyde (MDA) contents were measured to determine the degree of damage to cotton leaves according to the standard methods (Solarbio, Beijing, China).

## 5. Conclusions

In the present study, a comprehensive analysis of transcriptomic of PEG and salinity stresses was performed in cotton. The DEGs and co-expression analysis showed differences in the number of genes contained in each module. Most of the TFs belonged to the WGD events in the gene expansion analysis. Moreover, KEGG and GO analysis proved that the peptidase activity, transporter activity, and lipid metabolic processes were critical to cotton abiotic stresses. Several hub genes contained within network modules were associated with abiotic stresses. Moreover, qRT-PCR assays demonstrated that numerous hub genes were further proved to respond to the salt and PEG-induced drought stress, including *GhWRKY46*. As a TF, *GhWRKY46* plays its role at the nucleus. In addition, the VIGS assays and the measurement of MDA proved that the *GhWRKY46* plays a positive role in the salt and PEG-induced drought stress. These results provide valuable information for further research investigating the salt and drought tolerance in cotton and provide a new gene resource for future breeding.

## Figures and Tables

**Table 1:** Genes distribution in the co-expression modules.

Treat	Module Name	Gene Number	DEGs UP	DEGs DOWN	TF Prediction
PEG	blue	1187	109 (9.18%)	202 (17.02%)	133 (11.20%)
	brown	960	93 (9.68%)	112 (11.67%)	75 (7.81%)
	turquoise	2690	461 (17.14%)	196 (7.29%)	132 (4.91%)
	yellow	119	9 (7.56%)	15 (12.61%)	6 (5.04%)
Salt	blue	1359	109 (8.02%)	266 (19.57%)	97 (7.14%)
	brown	948	105 (11.08%)	124 (13.08%)	70 (7.38%)
	green	64	4 (6.25%)	14 (21.88%)	3 (4.69%)
	turquoise	1900	265 (13.95%)	141 (7.42%)	97 (5.11%)
	yellow	707	71 (10.04%)	105 (14.85%)	57 (8.06%)

**Table 2:** The prediction of the duplicated TFs in different modules.

Type	PEG		Salt		
	Blue	Turquoise	Brown	Blue	Turquoise
Singleton	0	0	0	1	0
Dispersed	1	1	2	3	2
Proximal	0	0	0	0	0
Tandem	3	3	2	0	0
WGD	132	128	66	93	95

## Data Availability

Not applicable.

## Supplementary Materials

The following supporting information can be downloaded at: https://www.mdpi.com/article/10.3390/ijms232012181/s1. References [74,75] are cited in the supplementary materials.

## References

[1] Anjum S.A., Xie X.Y., Wang L.C., Saleem M.F., Man C., Lei W. (2011). Morphological, physiological and biochemical responses of plants to drought stress. Afr. J. Agric. Res..

[2] Gray S.B., Brady S.M. (2016). Plant developmental responses to climate change. Dev. Biol..

[3] Zhu J.K. (2016). Abiotic Stress Signaling and Responses in Plants. Cell.

[4] Nakabayashi R., Saito K. (2015). Integrated metabolomics for abiotic stress responses in plants. Curr. Opin. Plant Biol..

[5] Kosova K., Vitamvas P., Urban M.O., Prasil I.T., Renaut J. (2018). Plant Abiotic Stress Proteomics: The Major Factors Determining Alterations in Cellular Proteome. Front. Plant Sci..

[6] Batista-Silva W., Heinemann B., Rugen N., Nunes-Nesi A., Araujo W.L., Braun H.P., Hildebrandt T.M. (2019). The role of amino acid metabolism during abiotic stress release. Plant Cell Environ..

[7] Hoang X.L.T., Nhi D.N.H., Thu N.B.A., Thao N.P., Tran L.P. (2017). Transcription Factors and Their Roles in Signal Transduction in Plants under Abiotic Stresses. Curr. Genom..

[8] Sun H., Hu M., Li J., Chen L., Li M., Zhang S., Zhang X., Yang X. (2018). Comprehensive analysis of NAC transcription factors uncovers their roles during fiber development and stress response in cotton. BMC Plant Biol..

[9] Chen P., Wei F., Cheng S., Ma L., Wang H., Zhang M., Mao G., Lu J., Hao P., Ahmad A. (2020). A comprehensive analysis of cotton VQ gene superfamily reveals their potential and extensive roles in regulating cotton abiotic stress. BMC Genom..

[10] Zhang J.B., Wang X.P., Wang Y.C., Chen Y.H., Luo J.W., Li D.D., Li X.B. (2020). Genome-wide identification and functional characterization of cotton (*Gossypium hirsutum*) MAPKKK gene family in response to drought stress. BMC Plant Biol..

[11] Yao D., Zhang X., Zhao X., Liu C., Wang C., Zhang Z., Zhang C., Wei Q., Wang Q., Yan H. (2011). Transcriptome analysis reveals salt-stress-regulated biological processes and key pathways in roots of cotton (*Gossypium hirsutum* L.). Genomics.

[12] Zhang J., Zhang P., Huo X., Gao Y., Chen Y., Song Z., Wang F., Zhang J. (2021). Comparative Phenotypic and Transcriptomic Analysis Reveals Key Responses of Upland Cotton to Salinity Stress During Postgermination. Front. Plant Sci..

[13] Wang D., Lu X., Chen X., Wang S., Wang J., Guo L., Yin Z., Chen Q., Ye W. (2020). Temporal salt stress-induced transcriptome alterations and regulatory mechanisms revealed by PacBio long-reads RNA sequencing in *Gossypium hirsutum*. BMC Genom..

[14] Zhao G., Song Y., Wang Q., Yao D., Li D., Qin W., Ge X., Yang Z., Xu W., Su Z. (2020). *Gossypium hirsutum* Salt Tolerance Is Enhanced by Overexpression of G. arboreum JAZ1. Front. Bioeng Biotechnol..

[15] Dou L., Sun Y., Li S., Ge C., Shen Q., Li H., Wang W., Mao J., Xiao G., Pang C. (2021). Transcriptomic analyses show that 24-epibrassinolide (EBR) promotes cold tolerance in cotton seedlings. PLoS ONE.

[16] Cheng G., Zhang L., Wang H., Lu J., Wei H., Yu S. (2020). Transcriptomic Profiling of Young Cotyledons Response to Chilling Stress in Two Contrasting Cotton (*Gossypium hirsutum* L.) Genotypes at the Seedling Stage. Int. J. Mol. Sci..

[17] Zhang X., Yao D., Wang Q., Xu W., Wei Q., Wang C., Liu C., Zhang C., Yan H., Ling Y. (2013). mRNA-seq analysis of the Gossypium arboreum transcriptome reveals tissue selective signaling in response to water stress during seedling stage. PLoS ONE.

[18] Cheng X., Zhao Y., Jiang Q., Yang J., Zhao W., Taylor I.A., Peng Y.L., Wang D., Liu J. (2019). Structural basis of dimerization and dual W-box DNA recognition by rice WRKY domain. Nucleic Acids Res..

[19] Chen J., Nolan T.M., Ye H., Zhang M., Tong H., Xin P., Chu J., Chu C., Li Z., Yin Y. (2017). Arabidopsis WRKY46, WRKY54, and WRKY70 Transcription Factors Are Involved in Brassinosteroid-Regulated Plant Growth and Drought Responses. Plant Cell.

[20] Jiang J., Ma S., Ye N., Jiang M., Cao J., Zhang J. (2017). WRKY transcription factors in plant responses to stresses. J. Integr. Plant Biol..

[21] Eulgem T., Rushton P.J., Robatzek S., Somssich I.E. (2000). The WRKY superfamily of plant transcription factors. Trends Plant Sci..

[22] Zhou X., Jiang Y., Yu D. (2011). WRKY22 transcription factor mediates dark-induced leaf senescence in Arabidopsis. Mol. Cells.

[23] Ding Z.J., Yan J.Y., Xu X.Y., Yu D.Q., Li G.X., Zhang S.Q., Zheng S.J. (2014). Transcription factor WRKY46 regulates osmotic stress responses and stomatal movement independently in Arabidopsis. Plant J..

[24] Shi W.Y., Du Y.T., Ma J., Min D.H., Jin L.G., Chen J., Chen M., Zhou Y.B., Ma Y.Z., Xu Z.S. (2018). The WRKY Transcription Factor GmWRKY12 Confers Drought and Salt Tolerance in Soybean. Int. J. Mol. Sci..

[25] Guo Q., Zhao L., Fan X., Xu P., Xu Z., Zhang X., Meng S., Shen X. (2019). Transcription Factor GarWRKY5 Is Involved in Salt Stress Response in Diploid Cotton Species (Gossypium aridum L.). Int. J. Mol. Sci..

[26] Ullah A., Sun H., Hakim, Yang X., Zhang X. (2018). A novel cotton WRKY gene, GhWRKY6-like, improves salt tolerance by activating the ABA signaling pathway and scavenging of reactive oxygen species. Physiol. Plant.

[27] Wang J., Wang L., Yan Y., Zhang S., Li H., Gao Z., Wang C., Guo X. (2021). GhWRKY21 regulates ABA-mediated drought tolerance by fine-tuning the expression of GhHAB in cotton. Plant Cell Rep..

[28] Wang N.N., Xu S.W., Sun Y.L., Liu D., Zhou L., Li Y., Li X.B. (2019). The cotton WRKY transcription factor (GhWRKY33) reduces transgenic Arabidopsis resistance to drought stress. Sci. Rep..

[29] Chu X., Wang C., Chen X., Lu W., Li H., Wang X., Hao L., Guo X. (2015). The Cotton WRKY Gene GhWRKY41 Positively Regulates Salt and Drought Stress Tolerance in Transgenic Nicotiana benthamiana. PLoS ONE.

[30] Gu L., Ma Q., Zhang C., Wang C., Wei H., Wang H., Yu S. (2019). The Cotton GhWRKY91 Transcription Factor Mediates Leaf Senescence and Responses to Drought Stress in Transgenic Arabidopsis thaliana. Front. Plant Sci..

[31] Gu L., Li L., Wei H., Wang H., Su J., Guo Y., Yu S. (2018). Identification of the group IIa WRKY subfamily and the functional analysis of GhWRKY17 in upland cotton (*Gossypium hirsutum* L.). PLoS ONE.

[32] Gu L., Dou L., Guo Y., Wang H., Li L., Wang C., Ma L., Wei H., Yu S. (2019). The WRKY transcription factor GhWRKY27 coordinates the senescence regulatory pathway in upland cotton (*Gossypium hirsutum* L.). BMC Plant Biol..

[33] Wang N.N., Li Y., Chen Y.H., Lu R., Zhou L., Wang Y., Zheng Y., Li X.B. (2021). Phosphorylation of WRKY16 by MPK3-1 is essential for its transcriptional activity during fiber initiation and elongation in cotton (*Gossypium hirsutum*). Plant Cell.

[34] Li S., Zheng T., Zhuo X., Li Z., Li L., Li P., Qiu L., Pan H., Wang J., Cheng T. (2020). Transcriptome profiles reveal that gibberellin-related genes regulate weeping traits in crape myrtle. Hortic. Res..

[35] Lu C., Pu Y., Liu Y., Li Y., Qu J., Huang H., Dai S. (2019). Comparative transcriptomics and weighted gene co-expression correlation network analysis (WGCNA) reveal potential regulation mechanism of carotenoid accumulation in Chrysanthemum x morifolium. Plant Physiol. Biochem..

[36] Wang J.-l., Zhang Y., Pan X.-D., Du J.-J., Ma L.-M., Guo X.-Y. (2019). Discovery of leaf region and time point related modules and genes in maize (Zea mays L.) leaves by Weighted Gene Co-expression Network analysis (WGCNA) of gene expression profiles of carbon metabolism. J. Integr. Agric..

[37] Peng H., He X., Gao J., Ma H., Zhang Z., Shen Y., Pan G., Lin H. (2015). Transcriptomic changes during maize roots development responsive to Cadmium (Cd) pollution using comparative RNAseq-based approach. Biochem. Biophys. Res. Commun..

[38] Zou X., Liu A., Zhang Z., Ge Q., Fan S., Gong W., Li J., Gong J., Shi Y., Tian B. (2019). Co-Expression Network Analysis and Hub Gene Selection for High-Quality Fiber in Upland Cotton (*Gossypium hirsutum*) Using RNA Sequencing Analysis. Genes.

[39] Tahmasebi A., Ashrafi-Dehkordi E., Shahriari A.G., Mazloomi S.M., Ebrahimie E. (2019). Integrative meta-analysis of transcriptomic responses to abiotic stress in cotton. Prog. Biophys. Mol. Biol..

[40] Wang Y., Liu J., Zhao G., Geng Z., Qi H., Dou H., Zhang H. (2020). Dynamic transcriptome and co-expression network analysis of the cotton (*Gossypium hirsutum*) root response to salinity stress at the seedling stage. Acta Physiol. Plant..

[41] Xu Y., Magwanga R.O., Jin D., Cai X., Hou Y., Juyun Z., Agong S.G., Wang K., Liu F., Zhou Z. (2020). Comparative transcriptome analysis reveals evolutionary divergence and shared network of cold and salt stress response in diploid D-genome cotton. BMC Plant Biol..

[42] You Q., Zhang L., Yi X., Zhang K., Yao D., Zhang X., Wang Q., Zhao X., Ling Y., Xu W. (2016). Co-expression network analyses identify functional modules associated with development and stress response in Gossypium arboreum. Sci. Rep..

[43] You Q., Xu W., Zhang K., Zhang L., Yi X., Yao D., Wang C., Zhang X., Zhao X., Provart N.J. (2017). ccNET: Database of co-expression networks with functional modules for diploid and polyploid Gossypium. Nucleic Acids Res..

[44] Wang D., Fan W., Guo X., Wu K., Zhou S., Chen Z., Li D., Wang K., Zhu Y., Zhou Y. (2020). MaGenDB: A functional genomics hub for Malvaceae plants. Nucleic Acids Res..

[45] Abdelraheem A., Esmaeili N., O’Connell M., Zhang J.F. (2019). Progress and perspective on drought and salt stress tolerance in cotton. Ind. Crops Prod..

[46] Hu Y., Chen J., Fang L., Zhang Z., Ma W., Niu Y., Ju L., Deng J., Zhao T., Lian J. (2019). Gossypium barbadense and *Gossypium hirsutum* genomes provide insights into the origin and evolution of allotetraploid cotton. Nat. Genet.

[47] Dong Y., Hu G., Yu J., Thu S.W., Grover C.E., Zhu S., Wendel J.F. (2020). Salt-tolerance diversity in diploid and polyploid cotton (Gossypium) species. Plant J..

[48] Fang L., Zhao T., Hu Y., Si Z., Zhu X., Han Z., Liu G., Wang S., Ju L., Guo M. (2021). Divergent improvement of two cultivated allotetraploid cotton species. Plant Biotechnol. J..

[49] Lan Y., Sun R., Ouyang J., Ding W., Kim M.J., Wu J., Li Y., Shi T. (2021). AtMAD: Arabidopsis thaliana multi-omics association database. Nucleic Acids Res..

[50] Ran X., Zhao F., Wang Y., Liu J., Zhuang Y., Ye L., Qi M., Cheng J., Zhang Y. (2020). Plant Regulomics: A data-driven interface for retrieving upstream regulators from plant multi-omics data. Plant J..

[51] Zhao W., Wang J., He X., Huang X., Jiao Y., Dai M., Wei S., Fu J., Chen Y., Ren X. (2004). BGI-RIS: An integrated information resource and comparative analysis workbench for rice genomics. Nucleic Acids Res..

[52] Zhu T., Liang C., Meng Z., Sun G., Meng Z., Guo S., Zhang R. (2017). CottonFGD: An integrated functional genomics database for cotton. BMC Plant Biol..

[53] Orsini F., D’Urzo M.P., Inan G., Serra S., Oh D.H., Mickelbart M.V., Consiglio F., Li X., Jeong J.C., Yun D.J. (2010). A comparative study of salt tolerance parameters in 11 wild relatives of Arabidopsis thaliana. J. Exp. Bot..

[54] Zhang L., Wu S., Chang X., Wang X., Zhao Y., Xia Y., Trigiano R.N., Jiao Y., Chen F. (2020). The ancient wave of polyploidization events in flowering plants and their facilitated adaptation to environmental stress. Plant Cell Environ..

[55] Qiao X., Li Q., Yin H., Qi K., Li L., Wang R., Zhang S., Paterson A.H. (2019). Gene duplication and evolution in recurring polyploidization-diploidization cycles in plants. Genome Biol..

[56] Wu S., Han B., Jiao Y. (2020). Genetic Contribution of Paleopolyploidy to Adaptive Evolution in Angiosperms. Mol. Plant.

[57] Liu X.X., Zhang J.G., Ying L., Rao G.D. (2020). Metabolome and Transcriptome Analyses Reveal Tissue-Specific Variations in Gene Expression and Metabolites of Olive. J. Plant Biol..

[58] Xiong X., Sun S., Li Y., Zhang X., Sun J., Xue F. (2019). The cotton WRKY transcription factor GhWRKY70 negatively regulates the defense response against Verticillium dahliae. Crop J..

[59] Morales M., Munne-Bosch S. (2019). Malondialdehyde: Facts and Artifacts. Plant Physiol..

[60] Bolger A.M., Lohse M., Usadel B. (2014). Trimmomatic: A flexible trimmer for Illumina sequence data. Bioinformatics.

[61] Kim D., Langmead B., Salzberg S.L. (2015). HISAT: A fast spliced aligner with low memory requirements. Nat. Methods.

[62] Li H., Handsaker B., Wysoker A., Fennell T., Ruan J., Homer N., Marth G., Abecasis G., Durbin R. (2009). 1000 Genome Project Data Processing Subgroup. The Sequence Alignment/Map format and SAMtools. Bioinformatics.

[63] Liao Y., Smyth G.K., Shi W. (2014). featureCounts: An efficient general purpose program for assigning sequence reads to genomic features. Bioinformatics.

[64] Robinson M.D., McCarthy D.J., Smyth G.K. (2010). edgeR: A Bioconductor package for differential expression analysis of digital gene expression data. Bioinformatics.

[65] Langfelder P., Horvath S. (2008). WGCNA: An R package for weighted correlation network analysis. BMC Bioinform..

[66] Chin C.H., Chen S.H., Wu H.H., Ho C.W., Ko M.T., Lin C.Y. (2014). cytoHubba: Identifying hub objects and sub-networks from complex interactome. BMC Syst. Biol..

[67] Shannon P., Markiel A., Ozier O., Baliga N.S., Wang J.T., Ramage D., Amin N., Schwikowski B., Ideker T. (2003). Cytoscape: A software environment for integrated models of biomolecular interaction networks. Genome Res..

[68] Huerta-Cepas J., Szklarczyk D., Forslund K., Cook H., Heller D., Walter M.C., Rattei T., Mende D.R., Sunagawa S., Kuhn M. (2016). eggNOG 4.5: A hierarchical orthology framework with improved functional annotations for eukaryotic, prokaryotic and viral sequences. Nucleic Acids Res..

[69] Yu G., Wang L.G., Han Y., He Q.Y. (2012). clusterProfiler: An R package for comparing biological themes among gene clusters. OMICS.

[70] Lescot M., Dehais P., Thijs G., Marchal K., Moreau Y., Van de Peer Y., Rouze P., Rombauts S. (2002). PlantCARE, a database of plant cis-acting regulatory elements and a portal to tools for in silico analysis of promoter sequences. Nucleic Acids Res..

[71] Sun H., Wang J., Gong Z., Yao J., Wang Y., Xu J., Yuan G.C., Zhang Y., Shao Z. (2018). Quantitative integration of epigenomic variation and transcription factor binding using MAmotif toolkit identifies an important role of IRF2 as transcription activator at gene promoters. Cell Discov..

[72] Wang Y., Tang H., Debarry J.D., Tan X., Li J., Wang X., Lee T.H., Jin H., Marler B., Guo H. (2012). MCScanX: A toolkit for detection and evolutionary analysis of gene synteny and collinearity. Nucleic Acids Res..

[73] Livak K.J., Schmittgen T.D. (2001). Analysis of relative gene expression data using real-time quantitative PCR and the 2(-Delta Delta C(T)) Method. Methods.

[74] Wang M., Wang Q., Zhang B. (2013). Evaluation and selection of reliable reference genes for gene expression under abiotic stress in cotton (*Gossypium hirsutum* L.). Gene.

[75] Lu K., Li T., He J., Chang W., Zhang R., Liu M., Yu M., Fan Y., Ma J., Sun W. (2018). qPrimerDB: A thermodynamics-based gene-specific qPCR primer database for 147 organisms. Nucleic Acids Res..

