# Supplementary material for: A Comprehensive Gene Co-Expression Network Analysis Reveals a Role of GhWRKY46 in Responding to Drought and Salt Stresses
Source: Int J Mol Sci. 2022 Oct 12;23(20):12181. doi: 10.3390/ijms232012181 (PMC9603583; doi:10.3390/ijms232012181)
Supplement: Supplementary file 1 [file ijms-23-12181-s001.zip › captions.pdf]

Figure S1. Veen analysis of the DEGs in PEG and salinity stresses. Veen analysis at different stages at 1h (a), 3h (b), 6h (c), 12h (d), and 24h (e) in drought and salinity stresses. Veen analysis of Differential Up- and Down-regulated Genes in Salt Treatment and Drought Treatment (f);

Figure S2. Heatmaps of the correlation between modules and traits under PEG (a) and salinity (b) treatments;

Figure S3. Phylogenetic, gene structure, and sequence analysis of *GhWRKY46*. (a) The phylogenetic tree of the *GhWRKY46*. (b) Gene structure of *GhWRKY46*. (c) Sequence alignment of the deduced proteins with its homologous proteins *AtWRKY46* (*AT2G46400*), and *TcWRKY46* (XP_*007051596*)

Table S1. Summary of RNA-seq of the 32 tissues samples;

Table S2. Primers used in this study.;

Table S3. Summary and analysis of differentially expressed genes.

Table S4. The prediction of transcription factor through the PlantTFDB

Table S5. The distribution of TFs in cotton.

Table S6. The duplicated type of select genes in different modules. The 0 to 4 indicate the singleton, dispersed, proximal, tandem, WGD duplication type, respectively.

Table S7. qRT-PCR analysis of hub genes in PEG and salinity stresses.

Table S8. GO enrichment in Peg and salt treatment of different modules.

Table S9. *cis*-element prediction on the promoter of *GhWRKY45* co-expression interaction genes.
