# Supplementary figures and images for: A Comprehensive Gene Co-Expression Network Analysis Reveals a Role of GhWRKY46 in Responding to Drought and Salt Stresses
Source: Int J Mol Sci. 2022 Oct 12;23(20):12181. doi: 10.3390/ijms232012181 (PMC9603583; doi:10.3390/ijms232012181)

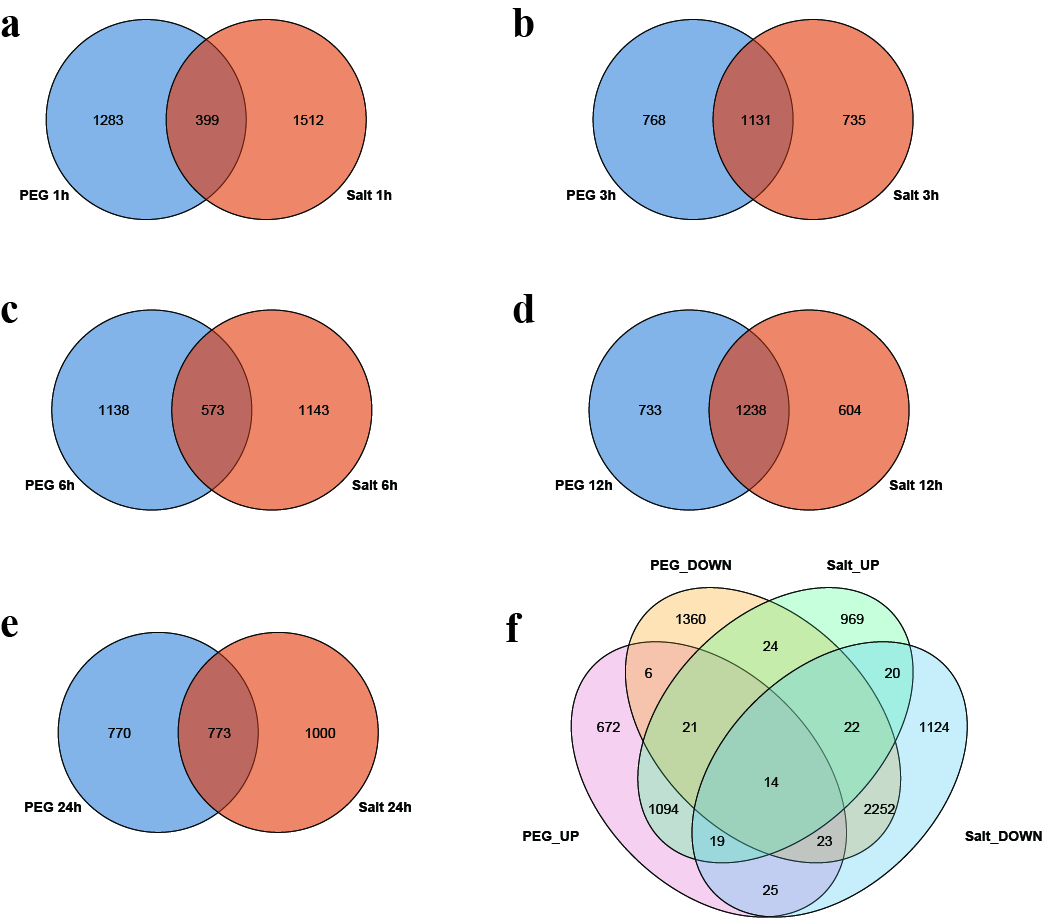

Supplement: Supplementary file 1 [file ijms-23-12181-s001.zip › Figure S1.tif]

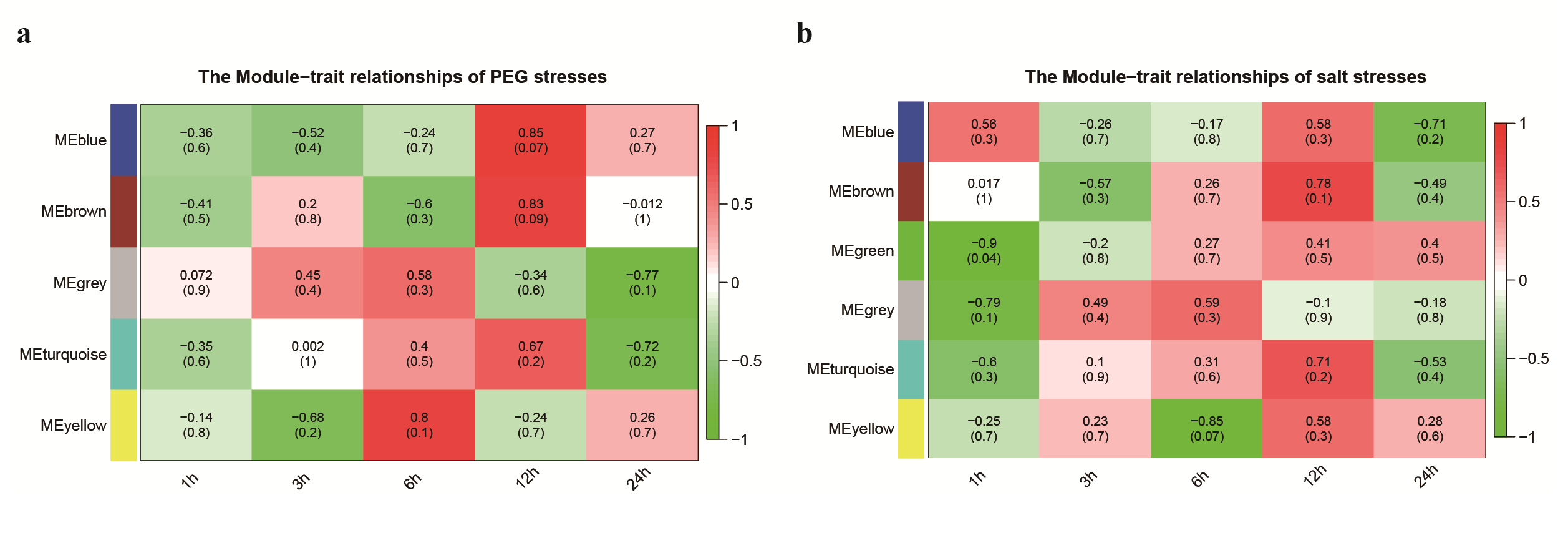

Supplement: Supplementary file 1 [file ijms-23-12181-s001.zip › Figure S2..tif]

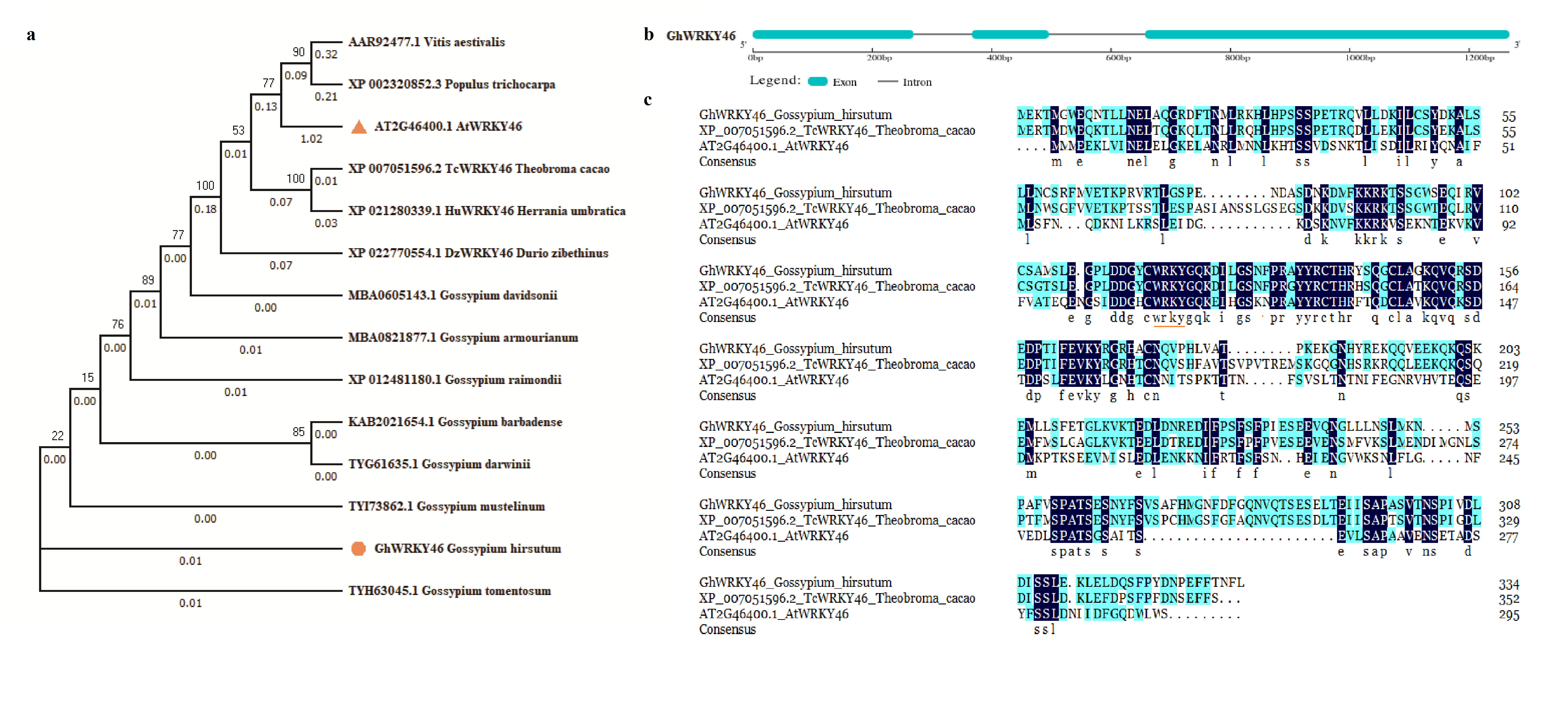

Supplement: Supplementary file 1 [file ijms-23-12181-s001.zip › Figure S3.tif]
